# Vildagliptin Enhances Differentiation of Insulin Producing Cells from Adipose-Derived Mesenchymal Stem Cells

**DOI:** 10.22074/cellj.2019.5542

**Published:** 2018-08-05

**Authors:** Samaneh Karimi, Jafar Ai, Layasadat Khorsandi, Darioush Bijan Nejad, Ghasem Saki

**Affiliations:** 1 Cell and Molecular Research Center, Faculty of Medicine, Ahvaz Jundishapur University of Medical Sciences, Ahvaz, Iran; 2 Tissue Engineering and Applied Cell Sciences, Department-School of Advanced Technologies in Medicine, Tehran University of Medical Sciences, Tehran, Iran

**Keywords:** Adipose Tissue, Insulin-Secreting Cells, Mesenchymal Stem Cells

## Abstract

**Objective:**

Type 1 diabetes is caused by destruction of beta cells of pancreas. Vildagliptin (VG), a dipeptidyl peptidase IV (DPP IV) inhibitor, is an anti-diabetic drug, which increases beta cell mass. In the present study, the effects of VG on generation of insulin-producing cells (IPCs) from adipose-derived mesenchymal stem cells (ASCs) is investigated.

**Materials and Methods:**

In this experimental study, ASCs were isolated and after characterization were exposed to differentiation media with or without VG. The presence of IPCs was confirmed by morphological analysis and gene expression (Pdx-1, Glut-2 and Insulin). Newport Green staining was used to determine insulin-positive cells. Insulin secretion under different concentrations of glucose was measured using radioimmunoassay method.

**Results:**

In the presence of VG the morphology of differentiated cells was similar to the pancreatic islet cells. Expression of Pdx-1, Glut-2 and Insulin genes in VG-treated cells was significantly higher than the cells exposed to induction media only. Insulin release from VG-treated ASCs showed a nearly 3.6 fold (P<0.05) increase when exposed to a high- glucose medium in comparison to untreated ASCs. The percentage of insulin-positive cells in the VG-treated cells was approximately 2.9-fold higher than the untreated ASCs.

**Conclusion:**

The present study has demonstrated that VG elevates differentiation of ASCs into IPCs. Improvement of this protocol may be used in cell therapy in diabetic patients.

## Introduction

Diabetes mellitus is one of the most common chronic diseases, with a progressively increasing number of people affected by this disease around the world (1). Type 1 diabetes is caused by the loss or destruction of beta cells. It is difficult to maintain optimal insulin dosage in diabetic patients, hence insulin administration does not completely prevent the conditions associated with diabetes. Therefore, transplantation of insulin-producing cells (IPCs) is potentially an ultimate cure for type 1 diabetes (2). Pancreatic beta cell failure is also involved in type 2 diabetes. It is well known that both beta cell function and mass decline progressively in type 2 diabetes (3). 

Numerous researchers have focused on generation IPCs. These cells may be derived from progenitor cells of the pancreas, bone marrow-derived mesenchymal stem cells, skin derived stem cells, adipose derived mesenchymal stem cells, pluripotent embryonic stem cells, and hepatic tissue (4-9). However, they have poor efficiency.

Glucagon-like peptide 1 (GLP-1) is produced in the intestine and secreted into the plasma in response to food intake. GLP-1 reduces gastric emptying time, decreases food intake and stimulates transcription of the proinsulin gene in beta cells. Hence, GLP-1 is considered as a therapeutic agent for type 2 diabetes. GLP-1 enhances the effects of glucose in stimulating insulin secretion from the beta cells. It reduces blood glucose concentration and stimulates insulin secretion in diabetic mice (10). In addition, GLP-1 increases the beta cell mass by stimulating the neogenesis and differentiation of ductal stem cells into endocrine cells (11, 12). On the other hand, GLP-1 is promptly degenerated by dipeptidyl peptidase IV (DPP IV). In the past decade DPP IV inhibitors have been progressively used for treatment of type 2 diabetes, including vildagliptin, sitagliptin, alogliptin, gemigliptin, saxagliptin, linagliptin and anagliptin (13). Several studies have examined the acute and chronic effects of DPP IV inhibitors on pancreatic islet and beta cell morphology in animals (14). Chronic administration of DPP IV inhibitors increased beta cell number via enhancing cell proliferation and preventing apoptosis (15). Vildagliptin (VG), a DPP IV inhibitor, covalently binds to the catalytic site of DPP IV, hence increasing GLP-1 levels (16). Foley et al. (17) have reported that VG significantly elevates secretory capacity of beta cells in type 2 diabetic patients. Akarte et al. (18) have shown that VG ameliorates GLP1 and stimulates beta cell proliferation in streptozotocin induced diabetes in rats. Duttaroy et al. (19) have reported that VG increases beta cell mass.

To date, most studies have focused on the effects of VG on hypoglycemia and insulin secretion capacity of beta cells in diabetic subjects. But, the effects of VG on differentiation of stem cells into beta cells have not been investigated. In this study, the effects of VG on differentiation of IPCs from rat ASCs was evaluated. 

## Materials and Methods

Healthy adult male Wistar rats (6-8 weeks old, 160180 g) were used in this experimental study. The rats were purchased from the Research Animal Center of Jundishapur University. This work was performed according to the guidelines of the institution’s Animal Ethics Committee (approval number: IR.AJUMS.REC. 1395.772). Epididymal fat pads were isolated under sterile conditions. The fat pads were exposed to collagenase (1.0 mg/ml in DMEM) for 20 minutes at 37°C. Then, the obtained homogenous cell suspension was centrifuged at 1200 rpm for 10 minutes. The obtained cell pellet was resuspended in DMEM and then cultured in 25 cm^2^ flasks. The ASCs were refed every 3 days and passaged when the confluency reached to approximately 80% (8).

### Characterization of adipose stem cells

Prior to cell treatments, the expression of cell surface markers of passage 3 ASCs, including CD34 (Santa Cruz, USA), CD90 (Santa Cruz, USA), CD29 (Abcam, USA), CD105 (Abcam, USA), CD45 (Abcam, USA) and CD73 (Abcam, USA), were analyzed by FACSCanto™ flow cytometer (Becton Dickinson, San Jose, CA, USA). At passage 3, osteogenic and adipogenic differentiation potentials of ASCs were also evaluated using appropriate induction media as previously described (8). Oil red O (Sigma-Aldrich, USA) and Alizarin red (Sigma-Aldrich, USA) staining were used to determine adipogenic and osteogenic potentials of the ASCs, respectively (5, 8). 

### Experimental design

For all experiments passage 3 ASCs were used. In experimental groups, ASCs were cultured in IPC induction medium with or without VG (Santa Cruz, USA). The control group was cultured in serum-free DMEM only. Induction of ASCs was performed in 3 steps (5, 8). In the first step, 100,000 cells were cultured for 48 hours in serum-free, high-glucose DMEM (25 mmol/L) containing 0.5 mmol/L 2-mercaptoethanol (Sigma-Aldrich, USA) and 10 ng/ml activin A (Sigma-Aldrich, USA). In the second step, the cells were cultured in medium containing 30 ng/ml fibroblast growth factor (FGF, Sigma-Aldrich, USA), 2 mmol/L L-glutamine (Sigma-Aldrich, USA), 20 ng/ml epidermal growth factor (EGF, Sigma-Aldrich, USA), 2% B27 (Invitrogen, USA), and 1% non-essential amino acids (Invitrogen, USA) for 8 days. Finally, in the third step, the cells were cultured in a different medium containing 10 mmol/L nicotinamide (Sigma-Aldrich, Karimi et al. USA), 2% B27 and 10 ng/ml betacellulin (Sigma- Aldrich, USA) for 8 days. In the VG group, 10 ng/ml VG were added to the differentiation medium at steps 2 and 3. For accuracy in VG addition throughout the study, a stock solution of 0.01 mg/ml VG/DMEM was prepared and stored at 4°C. Based on our pilot studies 1 µl of this stock solution was added to the cells as mentioned above.

### Immunofluorescent staining

Newport Green (NG, Invitrogen, USA) dye was used to determine insulin-containing cells. NG is a fluorescent molecule that has an affinity for zinc, which is necessary to form insulin granules in beta cells. The cells were fixed with 4 % paraformaldehyde (Sigma-Aldrich, USA) for 20 minutes and permeabilized with 0.1 % Triton X-100 (Sigma-Aldrich, USA) in phosphate buffered saline (PBS) for 10 minutes at room temperature. Cells were exposed to 25 µM NG in PBS for 30 minutes at 37°C. After washing in PBS, the cells were analyzed under a fluorescent microscope (Olympus, Japan) and percentage of NG-positive cells was determined (20).

### Real time polymerase chain reaction 

RNeasy Mini kit (Qiagen, Germany), was used to isolate RNA from the cultured cells. cDNA synthesis kit was used to generation cDNA from the isolated RNAs (Qiagen, Germany). The sequences for all primers are shown in Table 1. Polymerase chain reaction (PCR) amplification performed over 45 cycles using the Applied Biosystems™ 7500 Real-Time PCR System, and the following program: 95°C for 10 minutes, 95°C for 25 seconds, 5°C for 50 seconds and 60°C for 45 seconds. Data were analyzed using the 2^-ΔΔCT^ method. Expression values were corrected for the housekeeping gene GAPDH (5, 8). 

**Table 1:** Primer sequences


Gene	Primer sequence (5′-3′)

*Pdx-1*	F: AAACGGCACACACAAGGAGAA
	R: AGACCTGGCGCTTCACATG
*Glut-2*	F: CAGCTGTCTTGTGCTCTGCTTGT
	R: GCCGTCATGCTCACATAACTCA
*Ins*	F: TCTTCTACACACCCATGTCCC
	R: GGTGCAGCACTGATCCAG
*GAPDH*	F: ACCCAGAAGACTGTGGATGG
	R: TTCTAGACGGCAGGTCAGGT


### Radioimmunoassay

The cultured cells in all groups were exposed to glucose- free Krebs-Ringer bicarbonate (KRB, Sigma-Aldrich, USA) for 1 hour. Then, the cells of each group were divided in three groups and exposed to KRB containing glucose at the concentration of 5.56, 16.7 and 25 mmol/L for 1 hour, Insulin contents were determined using a RIA kit (Millipore, Germany) (5, 8).

### Statistical analysis

The data were analyzed using one-way ANOVA followed by Post hoc LSD test and were presented as the mean ± SD. P<0.05 was considered significant.

## Results

### Characterization of adipose stem cells

Passage 3 ASCs had a spindle-like morphology. Flow cytometry assessments showed high expression levels of CD90 (99.4%), CD29 (97.3%), CD105 (96.4%) and CD73 (83.3%), whereas significantly lower expression levels of CD34 and CD45 were observed. After the ASCs were cultured in adipogenic medium for 2 weeks, lipid droplets were observed in their cytoplasm detected by Oil-red O staining. On the other hand, osteogenic medium treatment of ASCs resulted in generation of mineral deposits as indicated by Alizarin red staining (data not shown).

### Morphology 

**Fig.1:**
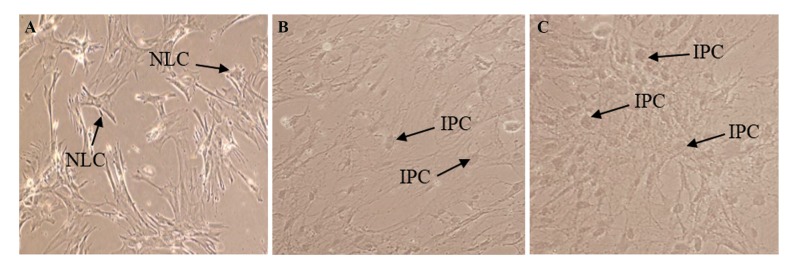
Morphological changes of ASCs. A. ASCs in only DMEM: various features including spherical, spindle fibroblast-like cells, and NLC are observed, B. ASCs in IPC induction medium in the absence of VG, and C. ASCs in IPC induction medium in the presence of VG. The IPCs show a round morphology (magnification: ×250). ASCs; Adipose-derived mesenchymal stem cells, NLC; Neuron-like cells, IPC; Insulin-producing cells, and VG; Vildagliptin.

In differentiation medium with VG, however, round cell morphology at a confluency similar to the pancreatic islet-like clusters was observed. Interestingly, in the cells cultured in differentiation medium without VG, the round morphology was less common. The control ASCs, at the first of experiment, had elongated morphology. The control ASCs appeared in various shapes including spherical, neuron-like cells or glial-like cells at the end of experiment (Fig .1).

### Immunofluorescence staining

**Fig.2:**
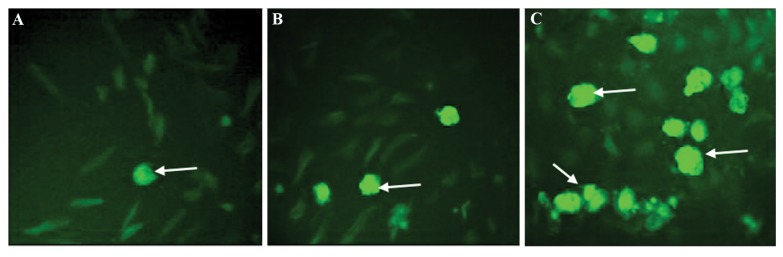
Immunofluorescence illustration of NG staining. A. Control ASCs in DMEM only, B. ASCs in IPC induction medium in the absence of VG, and C. ASCs in IPC induction medium in the presence of VG. Brilliant green indicating NG-positive cells (magnifications: ×400). NG; Newport green, ASCs; Adipose-derived mesenchymal stem cells, VG; Vildagliptin, and IPC; Insulin-producing cells.

**Fig.3:**
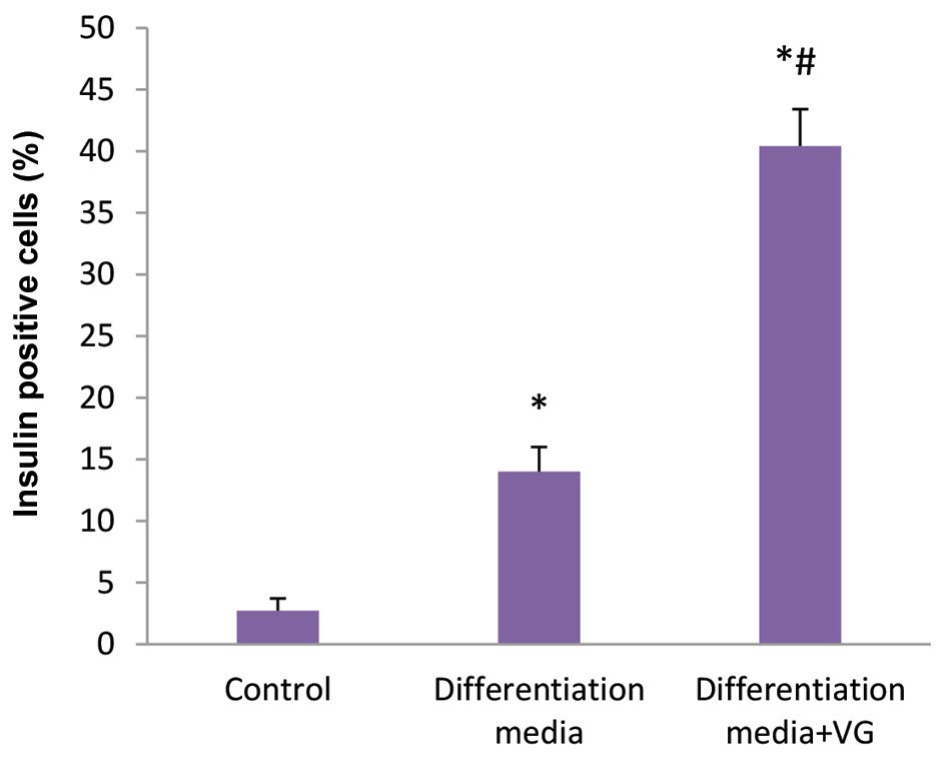
Percentage of NG-positive cells in various groups. Values are expressed as mean ± SD. ^*^; P<0.001, ^#^; P<0.001, * , #; Indicate comparison with the control and differentiation medium without VG, respectively, NG; Newport green, and VG; Vildagliptin.

Very few ASCs showed NG-positive staining in control group. The number of cells staining positive for NG was significantly higher in differentiation medium with VG, compared to the cells cultured in differentiation medium but in the absence of VG (P<0.001). In the control group only a few ASCs showed NG-positive staining (P<0.001). These data are illustrated in Figures 2 and 3. 

### Insulin release in response to glucose stimulation

**Fig.4:**
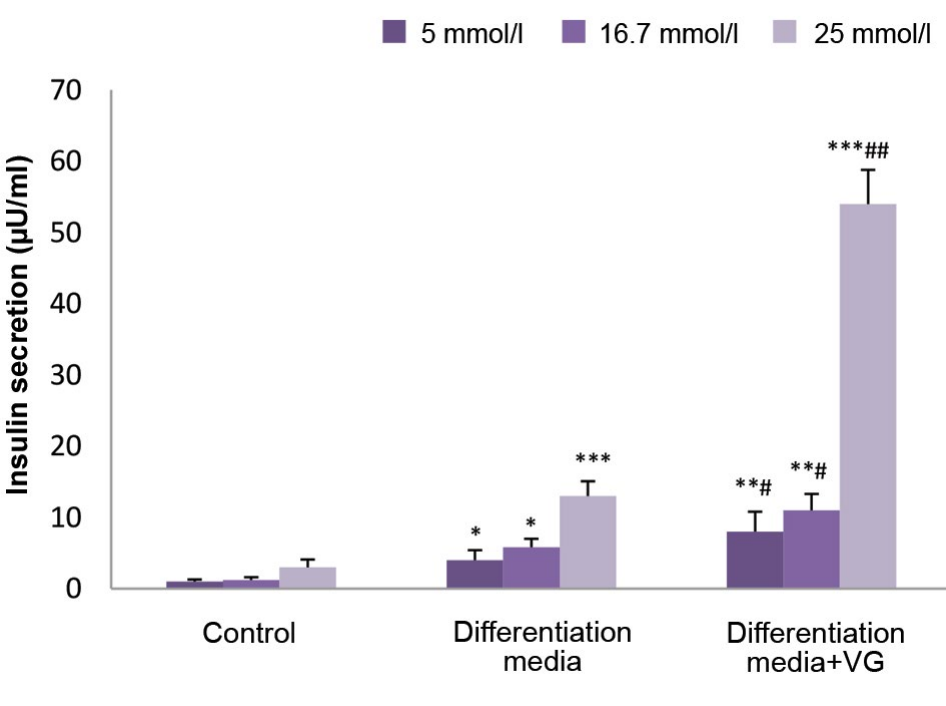
Insulin secretion in response to the low and high concentrations of glucose. Values are expressed as mean ± SD. *; P<0.01, **; P<0.01, ***; P<0.001, ^#^; P<0.05, ^##^; P<0.001, and ^* , #^; Indicate comparison with control and differentiation media without Vildagliptin (VG), respectively.

Insulin secretion at 5.56 mmol/L of glucose increased approximately 4.3 fold, and 5.6 fold at 25 mmol/L of glucose (a glucose challenge) (P<0.01) in the ASC- derived IPCs cultured in VG-free differentiation medium On the other hand, secretion of insulin was significantly elevated in VG-treated ASC-derived IPCs at 5.56 mmol/L of glucose (2 fold) and under a glucose challenge (4.2 fold), compared to the VG-untreated ASC-derived IPCs (P<0.01). In the control group, however, low levels of insulin in the absence or presence of the glucose challenge were observed (Fig .4). 

### Gene expression

**Fig.5:**
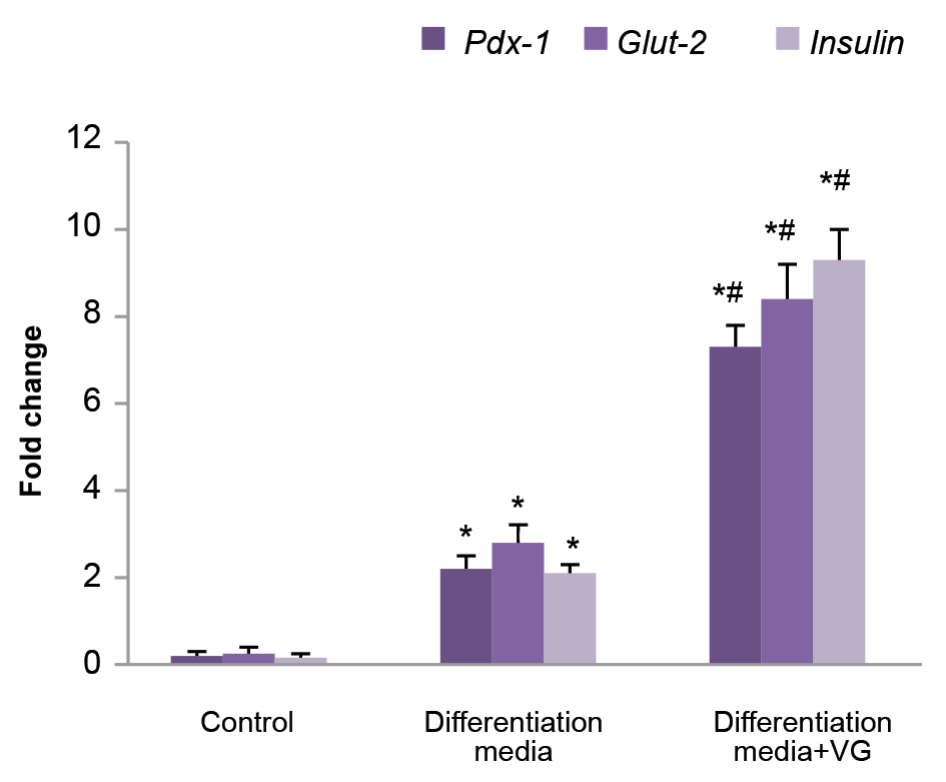
Gene expression in experimental and control groups. Expression normalized to the average of housekeeping gene (GAPDH). Values are expressed as mean ± SD. *; P<0.001, ^#^; P<0.001, ^* , #^; Indicate comparison with control and differentiation medium without Vildagliptin (VG), respectively.

Insulin (*Ins*), glucose transporter 2 (*Glut-2*) and *Pdx-1* showed low expression levels in the control cells. In comparison to the cells treated without VG, the expression of *Ins, Glut-2* and *Pdx-1* genes increased nearly 4.4 fold, 3 fold and 3.3 fold in the VG-treated IPCs (P<0.001), respectively (Fig .5).

## Discussion

The data presented here indicates that VG considerably enhances differentiation of ASCs into insulin- secreting cells. The presence of IPCs was confirmed by morphological evaluations, assessment of the expression pattern of islet-specific genes, and generation and secretion of insulin. The IPCs not only generated insulin, but also secreted insulin in response to glucose challenge. These responses were significantly higher in the presence of VG. 

We observed that VG significantly enhanced expression of *Pdx-1* gene in ASC-derived IPCs. Expression of *Pdx-1* is developmentally essential for both endocrine and exocrine portions of the pancreas, as it. *Pdx-1* regulates insulin gene transcription in response to glucose (21). The potential of *Pdx-1* to activate gene transcription is dependent on its ability to interact with other transcription factors (22). *Pdx-1* stimulates expression of several genes such as *Glut-2*, glucokinase (*GCK*) and *Ins*, which involves in maturation of beta cells (23). Miyagawa et al. (24) have shown that VG increases expression of insulin and *Pdx-1* genes, and elevates insulin secretion in a mice model of diabetes. 

In VG-treated cells, expression of other genes including *Insulin* and *Glut-2* was also significantly increased, which implied that the ASC-derived IPCs have undergone differentiation and maturation. In the beta cells, glucose uptake is regulated by *Glut-2*, which is critical for insulin secretion in response to glucose (25).

In addition, VG significantly enhanced insulin secretion in glucose challenge condition. The percentage of insulin-positive cells was elevated in the presence of VG compared to the VG-free group. These data revealed that VG effectively enhanced maturation of the ASC- derived IPCs. In a previous study, Foley et al. (17) have reported that VG significantly elevates secretory capacity of beta cells. Mari et al. (26) have also showed that VG improves beta cell function by increasing the insulin secretion capacity in diabetic patients. Utzschneider et al. (27) found that VG improves beta cell function and postprandial glycemia in patients with impaired fasting glucose. 

Previous studies have demonstrated that GLP-1 expands pancreatic beta cell mass by inducing proliferation and neogenesis of these cells (11, 12). Hence, VG, by suppression of DPP IV, may increase beta cell mass and consequently increase insulin secretion.

Duttaroy et al. (19) showed that VG significantly increased pancreatic beta cell mass in neonatal rats. In a preclinical study, VG and other DPP IV inhibitors were shown to expand beta cell mass (28). Shimizu et al. (29) have reported that VG protects beta cells against endoplasmic reticulum stress in C/EBPB transgenic mice.

In contrast, Gudipaty et al. (30) showed that sitagliptin, another DPP IV inhibitor, had no effect on the beta cell number. Hamamoto et al. (31) reported that VG enhanced beta cell mass by suppressing apoptosis, oxidative stress and endoplasmic reticulum stress, and induced proliferation and directly regulating beta cell differentiation in diabetic mice.

To our knowledge, this work is the first to study the effects of VG on generation of insulin-secreting cells. Almost all previous studies have reported VG effects on pancreas of diabetic patients or diabetic animal models. 

## Conclusion

The present work demonstrated that VG effectively enhanced differentiation of ASCs into the IPCs. Further *in vitro* and *in vivo* experiments are required to reveal the mechanisms, by which VG stimulates mesenchymal stem cell differentiation. 
